# Cervical Spine Involvement in Rheumatoid Arthritis: Does Joint Erosion Severity Correlate with Instability?

**DOI:** 10.5152/ArchRheumatol.2026.25127

**Published:** 2026-02-23

**Authors:** Mete Pekdiker, Sertaç Ketenci

**Affiliations:** 1 Division of Rheumatology, Department of Internal Medicine, Mustafa Kemal University, Hatay, Türkiye; 2 Division of Rheumatology, Department of Physical Therapy and Rehabilitation, Ondokuz Mayıs University, Samsun, Türkiye

**Keywords:** Atlantoaxial joint, joint ankylosis, radiography, rheumatoid arthritis, spine

## Abstract

**Background/Aims:**

Although numerous risk factors associated with cervical spine involvement (CSI) have been reported in the literature, the relationship between the severity of peripheral joint erosions and CSI has not yet been investigated. In this study, we aimed to investigate this relationship.

**Materials and Methods:**

Adult rheumatoid arthritis (RA) patients with a disease duration of more than 5 years were enrolled from 2 tertiary rheumatology clinics. Medical records were retrospectively reviewed. Neutral, open-mouth anteroposterior, full flexion, and full extension cervical spine radiographs, along with hand radiographs, were assessed independently by 2 blinded rheumatologists. “RA-type joint involvement (RJI)” was defined as presence of any erosion or joint space narrowing (JSN) as per the Modified Sharp Score (MSS). “Severe joint involvement (SJI)” was defined as presence of any erosion with a score of ≥ 3 points or JSN with a score of ≥ 4 points as per the MSS.

**Results:**

We enrolled 238 patients, 84.5% of whom were female, and the mean age was 58.1 ± 12.1 years. The frequency of CSI was 19.7% (n = 47), including anterior atlantoaxial subluxation (AAS) (42.3%), posterior AAS (7%), lateral AAS (22.5%), vertical subluxation (11.5%), and subaxial subluxation (12.7%). The CSI-positive group had longer lag time to diagnosis (*P *= .028), longer disease duration (*P* = .002), higher rates of RJI (*P* = .047), SJI (*P* < .001), and peripheral joint ankylosis (PJA) (*P* < .001) than CSI-negative group. In multivariate analysis, only PJA remained an independent predictor of CSI (*P* < .001). In addition, there was no significant difference in the frequency of interstitial lung disease and chronic knee/elbow arthritis between the CSI-positive and CSI-negative groups.

**Conclusion:**

To our knowledge, this is the first study to identify PJA as an independent risk factor for CSI. Therefore, RA patients having PJA should be carefully screened for CSI.

**Main Points**

- We found that 19.7% of patients with longstanding rheumatoid arthritis had cervical spine involvement (CSI).
- More than half of cervical spine lesions are of the non-anterior atlantoaxial subluxation type.
- Patients having CSI tended to have longer lag time to diagnosis, longer disease duration, more rheumatoid arthritis type joint involvement, more severe joint erosions, and more peripheral joint ankylosis.
- We identified for the first time peripheral joint ankylosis as the only independent predictive factor for (CSI).

## Introduction

Rheumatoid arthritis (RA) is an autoimmune, chronic, and multisystem inflammatory disease with a prevalence of 0.5-1%. Rheumatoid arthritis primarily affects peripheral synovial joints and leads to erosive arthritis resulting from bone and cartilage damage. Morbidity and mortality increase over the natural course of RA.^1^ In the early stages of RA, small joints of the hands and feet are generally involved; large joint involvement (LJI), such as the elbow, knee, shoulder, and hip, cervical spine involvement (CSI), and extra-articular manifestations (EAMs) are clinical features of established RA.^2^ Large joint involvement and EAMs predict worse radiological outcomes and poor prognosis in patients with RA.^2^^,^^3^

In the cervical spine, the occiput-C1 and C1-C2 joints contain synovial tissue, and chronic inflammation in these joints leads to pannus formation, bone erosions, ligamentous laxity, and spinal cord compression in patients with RA; however, the pathogenesis of CSI remains incompletely understood.^4^ In the medical literature, the frequency of CSI varies between 16% and 88% depending on the study design.^5^^,^^6^ Conventional radiography is the most widely used imaging modality for the assessment of CSI. Atlanto-axial subluxation (AAS) is the most common form of CSI, occurring at a rate of 65%; vertical subluxation (VS), also known as cranial settling or basilar invagination, is the second most common at 20%, and subaxial subluxation (SAS) is the third at 15%.^7^ The most common form of AAS is anterior AAS (aAAS), accounting for 75%, followed by lateral AAS (lAAS) and posterior AAS (pAAS), which occur at rates of 25% and 6-7%, respectively.^8^

There is no consensus regarding the monitoring CSI during the course of RA. The European League Against Rheumatism (EULAR) recommends performing cervical radiographs in patients with clinical suspicion of CSI; however, half of RA patients with CSI are asymptomatic.^9^^,^^10^ Additionally, CSI has a high prevalence and increases mortality in patients with RA.^7^^,^^11^ Therefore, some authors recommend routine radiological monitoring of CSI.^12^ Risk factors for CSI vary according to study design, but a meta-analysis showed that female gender, rheumatoid factor (RF) positivity, long-term corticosteroid treatment, younger age at disease onset, longer disease duration, peripheral joint erosions, higher disease activity, and elevated acute phase reactants such as C-reactive protein and erythrocyte sedimentation rate (ESR) are associated with increased risk of CSI in RA patients.^13^

Does the severity of peripheral joint erosion predict CSI? What are the frequency and associated factors of CSI in patients with established RA? The answers to these questions remain unclear. In this study, we aimed to clarify this issue.

## Materials and Methods

### Data Collection and Patient Selection

We analyzed patients with RA who were being followed in 2 tertiary outpatient rheumatology departments. Electronic medical records were retrospectively reviewed; demographic, clinical, laboratory, and treatment data were collected between March 2024 and March 2025. Inclusion criteria were fulfillment of the 2010 American College of Rheumatology (ACR)/EULAR RA classification criteria,^14^ aged 18 years or older, with a disease duration of ≥ 5 years, and continuous treatment with disease-modifying antirheumatic drugs (DMARDs). Exclusion criteria included the presence of inflammatory rheumatic diseases involving the cervical spine such as ankylosing spondylitis or psoriatic arthritis; nonrheumatic cervical spine diseases such as diffuse idiopathic skeletal hyperostosis (DISH) or ochronosis; history of trauma compromising cervical spine integrity; history of cervical spine surgery; and absence of specific imaging studies.

### Assessments

Anteroposterior (AP) hand x-rays taken within the last year were evaluated according to the Modified Sharp Score (MSS) system.^15^ “RA-type joint involvement (RJI)” was defined as the presence of any erosion or joint space narrowing (JSN) according to the MSS. “Severe joint involvement (SJI)” was defined as the presence of any erosion with a score of ≥3 points or JSN with a score of ≥4 points per the MSS. Peripheral joint ankylosis (PJA) was defined as JSN with a score of 4 points according to the MSS. The diagnosis of joint erosion (excluding the first interphalangeal and distal interphalangeal joints) was based on the EULAR definition as “interruption of the cortical bone.”^16^

Lateral cervical radiographs in neutral, full flexion, and extension positions, as well as open-mouth AP cervical radiographs, were used to assess CSI. Five cervical spine lesions were defined as CSI: aAAS, pAAS, lAAS, VS, and SAS. Anterior AAS was defined as “the distance between the anterior surface of the odontoid process of C2 and the posteroinferior aspect of the C1 tubercle (anterior atlanto-dental interval) exceeding 3 mm” on lateral flexion radiographs;^17^ pAAS as “the posterior aspect of the anterior arch of C1 lying behind the anterior part of the C2 vertebral body” on lateral extension radiographs;^18^ lAAS as “a shift of C1 over C2 by more than 2 mm on open-mouth cervical AP radiographs.”^8^ Vertical subluxation was defined as a displacement of more than 3 mm above the Chamberlain line (the distance between the hard palate and the posterior edge of the foramen magnum),^19^ and SAS as a horizontal displacement of any vertebra relative to an adjacent vertebra by more than 2 mm without osteophyte formation.^20^ Knee/elbow involvement was based on both clinical (having swollen and tender joints lasting longer than three months) and radiological evidence (presence of symmetrically joint narrow spacing). All radiological assessments were made by 2 rheumatologists (MP and SK) who were blinded to patient identities and clinical data. Radiological decision was made by full agreement. In cases of disagreement, x-rays were re-evaluated to reach a consensus, and then a final decision was made. Diagnosis of ILD was based on high-resolution computed tomography (HRCT) according to the American Thoracic Society/European Respiratory Society statement and Wijsenbeek M et al.^21^^,^^22^ High-resolution computed tomography scans performed within the past year were included in the evaluation.

Rheumatoid factor was measured by nephelometric assay, and serum samples with results ≥ 14 IU/mL were defined as positive. Anti-cyclic citrullinated peptide antibody2-IgG (anti-CCP) was measured by enzyme-linked immunosorbent assay (ELISA), and serum samples with results ≥ 5 U/mL were defined as positive. Lag time to diagnosis for RA was defined as the interval between the onset of arthritis and the initiation of conventional synthetic DMARD (csDMARD) therapy. Use and duration of biologic or targeted synthetic DMARDs (b/tsDMARDs) were also recorded.

### Statistical Analysis and Ethical Standards

In this study, we performed statistical analyses to evaluate the differences between groups with and without CSI. The dataset included various clinical and demographic variables, such as age, sex, smoking status, and biomarker levels. Additionally, we considered the seropositivity for RF and anti-CCP antibodies. Participants were divided into 2 groups based on the presence (CSI positive) or absence (CSI negative). Furthermore, we created additional groups for RF and anti-CCP titers, categorizing them as follows: RF titer < 100 IU/mL and ≥ 100 IU/mL; anti-CCP titer < 200 IU/mL and ≥ 200 IU/mL. We also defined a dual seropositive group where participants who tested positive for both RF and anti-CCP antibodies were classified as dual seropositive; a dual seronegative group, where participants who tested negative for both RF and anti-CCP antibodies were classified as dual seronegative.

For continuous variables, we used either the independent *t*-test or the Mann–Whitney *U*-test, depending on the normality of the data. Categorical variables were analyzed using chi-square tests or Fisher’s exact tests when expected frequencies were low. Descriptive statistics such as means and standard deviations (SD) were calculated for continuous variables, while counts and percentages were determined for categorical variables. The detailed results of our statistical analysis, including *P*-values, test statistics, and mean ± SD for continuous variables, are summarized in the tables. Significant differences were noted in several variables, which are highlighted and discussed in the results section. Logistic regression analysis was employed to identify independent variables associated with CSI. Initially, highly correlated variables within the dataset were identified, and variables with multicollinearity issues were removed based on variance inflation factor analysis. Variables showing significant differences were selected based on the results of *t*-tests, Mann–Whitney *U*-tests, and chi-square tests. Finally, significant variables were used to construct the logistic regression model. The results of the logistic regression analysis are presented in a table, including beta coefficients, 95% confidence intervals (CI), and *P*-values. To address the issue of multiple testing, the Bonferroni correction was applied.

The Ethics Committee of the Hatay Mustafa Kemal University where the study was conducted approved the study (date: 12/17/2025, decision number: 42-16). The study protocol complies with the ethical guidelines of the 1975 Declaration of Helsinki, previously approved by the institution’s human research committee.Informed consent was obtained from the patients.

## Results

We consecutively reviewed 728 patients with RA and enrolled 238 participants in the study. A total of 490 patients were excluded; 368 had a disease duration of less than 5 years, 102 had a history of irregular DMARD use or follow-up period, 12 patients lacked proper x-ray data, and 5 had DISH. 84.5% of the study population was female, and the mean age was 58.1 ± 12.1 years. The mean disease duration was 180 ± 98 months, and the mean lag time to diagnosis was 21.9 ± 27.9 months. Rheumatoid factor positivity was 70.2%, and the mean RF titer was 181.4 ± 409.9 IU/mL. Anti-CCP positivity was 72.2%, and the mean anti-CCP titer was 211.9 ± 506.1 U/mL. 93.7% of patients had RJI, 52.5% had SJI, and 34.4% had PJA. 32.3% of patients had chronic knee arthritis, and 15.5% had chronic elbow arthritis. The frequency of ILD was 11.7%. All patients had a history of corticosteroid therapy (>7.5 mg/day prednisolone) lasting longer than 3 months. Seventy-one percent of the patients were treated with biologic or targeted synthetic DMARDs (b/tsDMARDs), with rituximab being the most commonly used biologic agent (n = 68), followed by adalimumab (n = 28) and etanercept (n = 20). Twenty-six patients were using targeted synthetic DMARDs (tsDMARDs), such as tofacitinib or baricitinib.

**Figure 1.**
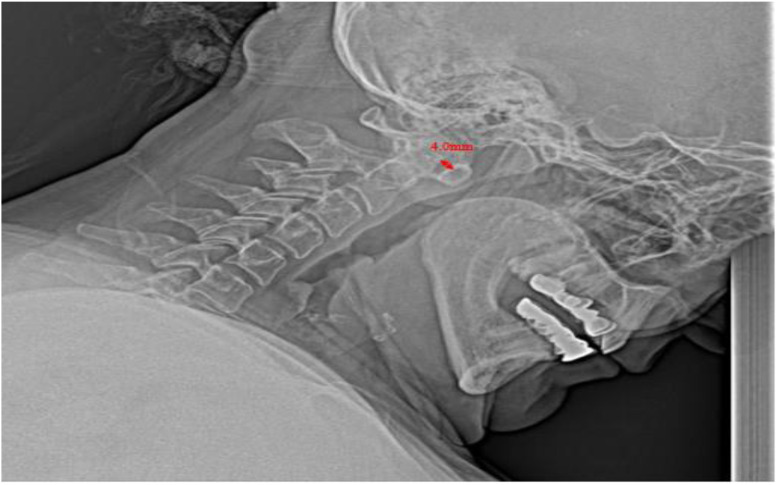
An example of anterior atlanto-axial subluxation from our study population; she had 12 years of disease duration, peripheral joint ankylosis, and dual seropositive serology.

**Figure 2.**
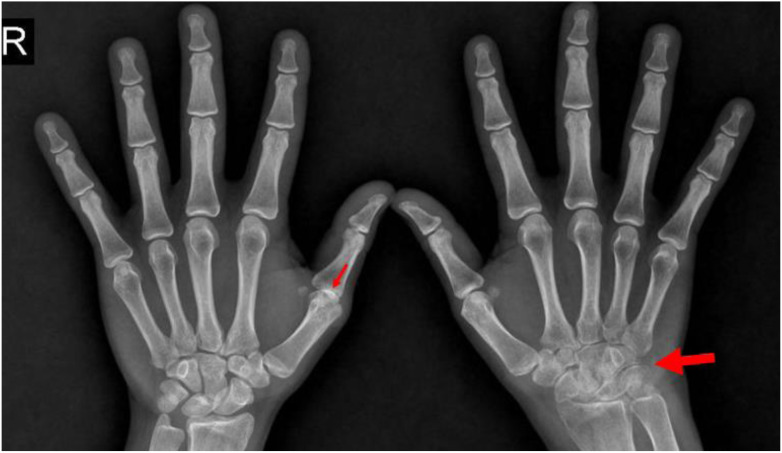
The hand x-ray of the above patient: Peripheral joint ankylosis in the first interphalangeal joint of the right hand, and joint space narrowing in multiple carpometacarpal, intercarpal, and radiocarpal joints of the left hand.

The frequency of CSI was 19.7% (n = 47). The distribution of CSI lesions was 64.8% for AAS, 15.5% for VS, and 12.7% for SAS. The total number of CSI lesions was 71; the most common lesion was aAAS, accounting for 42.5% (n = 30), while the rarest was pAAS at 7% (n = 5). One-third of our patients reported neck pain, but none had any neurological deficits. Table 1 shows demographic, laboratory, clinical, and treatment characteristics. Figure 1 shows an example of aAAS at our study population, and Figure 2 shows an example of PJA in the same patient in Figure 1.

The CSI-positive group had a significantly longer diagnostic delay (*P* = .028), longer disease duration (*P* = .002), more patients with RJI (*P* = .047), SJI (*P* < .001), and PJA (*P* < .001) than CSI-negative group. Rheumatoid factor and anti-CCP positivity and titers, LJI, and ILD were similar between the two groups. Table 2 shows the comparison of the two groups according to the CSI status. In the univariate analysis, disease duration, lag time to diagnosis, SJI, and RJI were identified as dependent predictive factors for CSI. Significant variables—including disease duration, lag time to diagnosis, SJI, and PJA—were included in the logistic regression model. The findings indicate that only PJA has a significant effect on the presence or absence of CSI (*P* = .001). Table 3 shows the univariate and multivariate analysis of significant variables.

## Discussion

In this multicenter observational study, we assessed the prevalence of CSI, its associated factors, and its relationship to late complications of established RA. We found that 19.7% of patients with longstanding RA had CSI. The CSI-positive patients tended to have a longer lag time to diagnosis, longer disease duration, and higher rates of RJI, SJI, and PJA. To our knowledge, this is the first study to identify PJA as an independent predictive factor for CSI in the medical literature. We also found no association between CSI and either LJI or ILD. We also screened for pAAS and lAAS, which are rarely reported in the medical literature; pAAS was detected in 2.1% and lAAS in 6.7% of patients.

Blom et al^5^ conducted a 12-year follow-up study on patients with RA and reported that 16% (n = 134) developed CSI. Patients with CSI had a higher number of peripheral joint erosions, and 7.3% had aAAS. Fujiwara K et al^23^ followed 162 RA patients over a mean duration of 10.2 years and reported a CSI frequency of 57%. The most common type was aAAS (46.7%), and 32 patients had mixed-type involvement (aAAS + VS). The incidence of CSI in the least erosive, more erosive, and mutilating groups (defined as <20, 20-40, and >40 eroded joints, respectively) was 39%, 83%, and 100%, respectively. Notably, patients in the mutilating group developed cervical lesions at an earlier stage (*P* < .01). Yurube T et al^24^ followed 140 RA patients without baseline CSI for 6 years and observed that 43.6% developed CSI during this period (AAS: 32.1%, VS: 11.4%, SAS: 16.4%, including some combinations). The authors identified mutilans-like hand deformities, progression to mutilating changes, and corticosteroid treatment as independent risk factors for CSI. Previous prospective studies have reported that a higher number of joint erosions is associated with the presence of CSI, but these studies did not investigate the relationship between erosion severity and CSI.^5^^,^^23^^,^^24^ Moreover, we investigated the radiological changes according to the MSS, also mutilans-like hand deformities defined according to the Larsen score system. Neva MH et al^25^ followed up 103 patients with RA during 8-13 years, finding the frequency of CSI to be 17%. They defined the early and extensive erosiveness of peripheral joints as independent predictors of CSI. Terashima Y et al^26^ defined the baseline mutilating changes, corticosteroid treatment, and previous joint surgery as independent predictors of severe CSI in a multicenter prospective cohort study. The most common finding in prospective studies is the association between peripheral joint erosions and CSI; however, this relationship is based on the presence and number of erosions. In previous studies, the severity of peripheral joint erosions has been associated with mutilating changes. This definition, according to the Larsen scoring system, refers to joint damage in which the original joint surfaces are lost, major bone deformities may be present, and the original bone outline is destroyed; these lesions receive a score of 5 in this system. In contrast, lesions defined as “severe abnormality” with a score of 4 correspond to the loss of joint space. In our study, the RJI represents a simpler and more easily applicable form of joint damage.^5^^,^^23^^-^^26^

Ahn JK et al^27^ conducted the largest study on CSI in Korea, including 1120 RA patients. They reported a CSI frequency of 28.6% and identified erosive peripheral joint disease and early RA diagnosis (< 45 years old) as independent predictive factors for CSI. Naranjo A et al^28^ conducted the second largest study on aAAS in Spain, involving 736 RA patients with a mean disease duration of 10 years. They found a CSI frequency of 12%, which increased over the course of RA. Early disease onset (before the age of 50 years) and a Larsen score above 50 were identified as important independent factors associated with aAAS.^28^ Studies with large patient populations focusing on CSI have also established a relationship between peripheral joint erosions and CS.^27^^,28^ Evaluating radiological scoring systems for hand deformities in RA is more complex and time-consuming than screening for PJA. Therefore, our findings may offer practical guidance for determining the optimal timing of CSI monitoring.

Alp G et al^29^ reported a frequency of aAAS of 10.4% in 240 RA patients with a mean disease duration of 8.5 years. They also identified anti-CCP titer as an independent predictive factor specifically for aAAS. Other forms of CSI were not detected in their study.^29^ Carotti M et al^30^ also reported that anti-CCP titer is an independent predictive factor for CSI. It is well known that both RF and anti-CCP are associated with increased joint erosions and higher disease activity in patients with RA. However, recent studies have shown that seronegative patients exhibit higher levels of inflammatory markers, more tender and swollen joints, and similar radiographic progression compared to seropositive patients.^31^^,^^32^ We also did not find any relationship between autoantibodies (RF and anti-CCP) and CSI.

There are few studies investigating the relationship between CSI and LJI or ILD. Alcala JM et al^33^ reported similar frequencies of ILD in CSI-positive and CSI-negative RA patients. Bettaieb H et al^34^ identified EAMs (including pulmonary involvement) as predictive factors for CSI; however, no association was found in multivariate analysis. Di Muzio et al^35^ demonstrated that EAMs (ILD and rheumatoid nodules) are associated with severe CSI in an MRI-based study. In our cohort, the CSI-positive group had a higher prevalence of ILD compared to the CSI-negative group, but this difference was not statistically significant (*P *= .13). Imagama S et al^36^ reported LJI as a predisposing factor for RA-related CSI. Also, bilateral shoulder involvement was defined as a risk factor for CSI in a study from Germany.^37^ In our cohort, the CSI-positive group had a higher frequency of LJI than the CSI-negative group; however, the difference was not statistically significant (*P* = .42 for knee involvement and *P* = .32 for elbow involvement). Kaito T et al^38^ reported a CSI frequency of 32% in RA patients (n = 151) treated with bDMARDs who had a disease duration of over 5 years, similar to our study. They identified disease duration and Steinbrocker stage as independent predictors of CSI. Additionally, 71% of our patients were using b/tsDMARDs, and 21.3% of these had CSI. A study from Japan showed that biologic agents do not reduce CSI incidence.^39^ Our results support these findings,^38^^,^^39^ as b/tsDMARD use was comparable between the CSI-positive and CSI-negative groups (*P* = .44).

The major limitations of our study include its retrospective design, absence of total MSS score, lack of intra- and interobserver reliability assessment, and the low sensitivity of the imaging method used. None of our patients had neurological deficits, and two-thirds were asymptomatic, resulting in no clear indication for cervical MRI in many cases. Although conventional radiography is the first-line imaging modality for detecting CSI, it has lower sensitivity compared to magnetic resonance imaging.^40^ Therefore, the frequency of CSI in our study is likely an underestimate of its true prevalence. While peripheral joint erosions are typical of early RA, joint ankylosis is a late-stage manifestation. Therefore, our results are not applicable to patients with early RA, and their practical value in terms of CSI prevention or monitoring in daily practice is limited. Another important limitation is that RA-related ILD was detected by HRCT only in symptomatic patients (e.g., dyspnea, dry cough) or those with suspicious findings on AP chest radiography, whereas most RA patients with ILD detected by HRCT are asymptomatic.^41^ Additionally, this study was conducted in 2 tertiary rheumatology centers where the proportion of patients receiving b/tsDMARDs was relatively high, which may introduce a selection bias.

In conclusion, we investigated the relationship between CSI and the severity of peripheral joint involvement in patients with RA. For the first time, we identified the presence of PJA as an independent predictive factor for RA-related CSI. Therefore, we suggest that rheumatologists screen for CSI when PJA is detected in RA cases. Prospective studies using more sensitive imaging modalities are needed to optimize the timing of CSI monitoring.

## Figures and Tables

**Table 1.** Demographic, Laboratory, Clinical, and Treatment Characteristics.

Total patient, n	238
Female sex, n (%)	201 (84.5)
Male sex, n (%)	37 (15.5)
Age, mean ± SD, years	58.1 ± 12.1
Lag time to diagnosis, mean ± SD, months	21.9 ± 27.9
Disease duration, mean ± SD, months	180 ± 98
Smoking (active or ex), n (%)	24 (10)
RF positivity, n (%)	167 (70.2)
RF titer, mean±SD (IU/mL)	181.4 ± 409.9
Anti-CCP positivity, n (%)	172 (72.2)
Anti-CCP titer, mean±SD, (U/mL)	211.9 ± 506.1
Patients with RJI, n (%)	223 (93.7)
Patients with SJI, n (%)	125 (52.5)
Patients with peripheral joint ankylosis, n (%)	82 (34.4)
Interstitial lung disease, n (%)	28 (11.8)
Chronic knee arthritis, n (%)	77 (32.3)
Chronic elbow arthritis, n (%)	37 (15.5)
b/tsDMARD use, n (%)	169 (71)
b/tsDMARD duration, mean ± SD, months	71 ± 47
Patients with CSI, n (%)	47 (19.7)
Total count of CSI lesions	71
Anterior AAS, n (%)	30 (42.3)
Posterior AAS, n (%)	5 (7)
Lateral AAS, n (%)	16 (22.5)
Vertical subluxation, n (%)	11 (15.5)
Subaxial subluxation, n (%)	9 (12.7)
Patients with mixed type CSI, n (%)	14 (29.8)
Atlanto-dental interval, mean ± SD, mm	5.6 ± 2

AAS, atlantoaxial subluxation; anti-CCP, anti-cyclic citrullinated peptide antibody-2; b/tsDMARD, biologic or target synthetic disease modifying anti-rheumatic drug; CSI, cervical spine involvement; RF, rheumatoid factor; RJI, rheumatoid arthritis-type joint involvement; SJI, severe joint involvement.

**Table 2.** Comparison of pPatients with and Without Cervical Spine Involvement.

**Variable**	**CSI+ group** **(n = 47)**	**CSI− group** **(n = 191)**	*P*-value
Female sex, n (%)	43 (91.5)	158 (82.7)	.179
Age, mean ± SD, years	59.2 ± 11.5	57.8 ± 12.3	.497
Lag time to diagnosis, mean ± SD, months	31.6 ± 34.1	19.6 ± 25.7	**.028**
Disease duration, mean ± SD, months	222.9 ± 103.9	170 ± 94.5	**.002**
Smoking, n (%)	8 (17)	48 (25.1)	.326
Patients with RJI, n (%)	47 (100)	176 (92.1)	**.047**
Patients with SJI, n (%)	36 (76.6)	89 (46.6)	**<.001**
Patients with PJA, n (%)	33 (70.2)	49 (25.7)	**<.001**
RF positivity, n (%)	38 (80.9)	129 (67.5)	.108
RF titer, mean ± SD, (IU/mL)	174.3 ± 205.2	279.5 ± 521.8	.358
RF titer ≥100 IU/mL, n (%)	17 (36.2)	72 (37.7)	.309
Anti-CCP positivity, n (%)	38 (80.9)	134 (70.2)	.199
Anti-CCP titer, mean ± SD, (U/mL)	415.4 ± 1158.9	257.1 ± 217.2	.703
Anti-CCP titer ≥200 IU/mL, n (%)	18 (38.3)	77 (40.3)	.358
Dual seropositivity, n (%)	35 (74.5)	120 (62.8)	.184
Dual seronegativity, n (%)	6 (12.8)	48 (25.1)	.105
Interstitial lung disease, n (%)	9 (19.1)	19 (9.9)	.133
Chronic knee arthritis, n (%)	18 (38.3)	59 (30.9)	.425
Chronic elbow arthritis, n (%)	10 (21.3)	27 (14.1)	.324
b/tsDMARD use, n (%)	36 (76.6)	133 (69.6)	.445
Duration of b/tsDMARD, mean ± SD, months	76 ± 51	70 ± 46	.457

anti-CCP, cyclic citrullinated peptide antibody-2; b/tsDMARD, biologic or target synthetic disease modifying anti-rheumatic drug; PJA, peripheral joint ankylosis; RF, rheumatoid factor; RJI, rheumatoid arthritis type joint involvement; SD, standard deviation; SJI, severe joint involvement. Values with *P* < .05 were considered statistically significant and are presented in bold.

**Table 3.** Logistic Regression Analysis to Identify Independent Variables Associated with Cervical Spine Involvement

**Univariate Logistic Regression**			**Multivariate Logistic Regression**		
**Variable**	**OR (95% CI)**	*P*-value	**Variable**	**OR (95% CI)**	*P*-value
Disease duration	1.005 (1.002- 1.008)	**.001**	Disease duration	1.003 (0.999-1.006)	.147
Lag time to diagnosis	1.013 (1.003-1.023)	**.011**	Lag time to diagnosis	1,007 (0.995-1.019)	.237
SJI	3.751 (1.803-7.804)	**<.001**	SJI	0.616 (0.162-2.349)	.478
PJA	6.831 (3.377-13.817)	**<.001**	PJA	7.936 (2.242-28.086)	**.001**

CI, confidence interval; OR, odds ratio; PJA, peripheral joint ankylosis; SJI, severe joint involvement. Values with *P* < .05 were considered statistically significant and are presented in bold.

## Data Availability

The data that support the findings of this study are available on request from the corresponding author.
